# Screening of Rubella Virus, Cytomegalovirus, Hepatitis B Virus, Hepatitis C Virus, HIV, Syphilis, and *Toxoplasma gondii* Antibodies in Pregnant Women

**DOI:** 10.3390/v18020206

**Published:** 2026-02-05

**Authors:** Fatih Mehmet Akıllı, Fatih Demir, Taylan Onat

**Affiliations:** 1 Department of Medical Microbiology, Sincan Training and Research Hospital, Health Sciences University, Ankara 34668, Turkey; 2 Department of Medical Microbiology, Aksaray Training and Research Hospital, Aksaray 68200,Turkey; 3 Department of Obstetrics and Gynecology, Sincan Training and Research Hospital, Health Sciences University, Ankara 34668, Turkey; fatihdemir28@gmail.com (F.D.); taylan.onat@inonu.edu.tr (T.O.)

**Keywords:** congenital infections, epidemiology, prenatal screening, public health, seroprevalence, TORCH, VDRL

## Abstract

TORCH pathogens are often asymptomatic in healthy adults but can cause foetal death when transmitted during pregnancy; therefore, accurate regional data are essential for screening. This study aimed to determine first-trimester TORCH seropositivity and to inform the development of hospital-based and national screening algorithms. This study analysed test results from 7481 pregnant women aged 15–49 years who participated between January 2020 and December 2024. TORCH serological results obtained using the MAGLUMI X3/X6 system (Snibe, Shenzhen, China) were analysed with Statistical Package for the Social Sciences. Anti-HCV positivity was 0.12% (9/7166), anti-*Toxoplasma gondii* IgG positivity was 16.5% (1027/6204), anti-rubella IgG positivity was 95.5% (5809/6080), and anti-CMV IgG positivity was 98.69% (6130/6211). Syphilis seropositivity among pregnant women was 0.2% (13/4991). Significant differences by age groups (15–24, 25–34, and >35 years) were observed for rubella IgG (*p* < 0.001), *T. gondii* IgG (*p* < 0.001), and HBsAg positivity (*p* = 0.009). This study investigated TORCH seropositivity among pregnant women in our hospital region and underscores the need for targeted public health initiatives to reduce the risk of congenital infections. It recommends systematic first-trimester assessment of TORCH exposure, standardized data recordings, the establishment of national screening programmes, and careful consideration of testing costs.

## 1. Introduction

The acronym TORCH refers to a group of pathogens—*Toxoplasma gondii*, Other infections (including *Treponema pallidum* (*T.* *pallidum*), hepatitis B virus (HBV), human immunodeficiency virus (HIV), hepatitis C virus (HCV), parvovirus B19, varicella zoster virus (VZV)), rubella, cytomegalovirus (CMV), and herpes simplex virus (HSV)—and was first defined in 1971 by Nahmias et al. TORCH infections can occur at any age and are often asymptomatic. However, infection during the first trimester of pregnancy carries a risk of vertical transmission to the foetus [1,2,3,4,5,6], leading to adverse outcomes such as spontaneous abortion, foetal death, and severe neurological sequelae, including hydrocephalus, intracranial calcifications, chorioretinitis, and blindness. Congenital infections account for up to 3% of all birth defects. Infections are often asymptomatic when acquired in the second or third trimester of pregnancy; nevertheless, delayed manifestation may occur years later, including hearing impairment and neuropsychomotor developmental delay. Incidence varies with factors such as nutrition and hygiene, animal contact, socioeconomic status, and environmental conditions [1,2,3,4,5,6,7,8,9].

Although TORCH agents are globally prevalent and readily diagnosed, no clear consensus on routine screening exists for these infections during pregnancy [10,11]. National prenatal care guidelines in our country do not recommend universal first-trimester TORCH screening but screening for HBsAg, syphilis, HIV, and rubella [11,12,13]. These infections are not recommended for routine first-trimester screening by the American College of Obstetricians and Gynecologists or the World Health Organization (WHO) [14,15], although some countries include them in routine prenatal screening programmes [15]. Toxoplasmosis, rubella, and CMV are initially diagnosed by measuring IgM and IgG antibodies using ELISA [15].

HBV is vaccine-preventable, and both HBV and HCV can cause acute hepatitis, cirrhosis, and hepatocellular carcinoma [16]. Globally, an estimated 257 million people are infected with HBV. The WHO estimates that chronic HCV affects approximately 58 million people worldwide, causing approximately 1.5 million new infections and 290,000 deaths annually [16,17]. Syphilis, caused by *T. pallidum* subsp. *pallidum*, can be transmitted transplacentally, sexually, or through blood transfusions and may lead to serious foetal complications during pregnancy [18,19].

Although studies have been conducted in other regions of our country, data are lacking for the location of our hospital. Therefore, this study aimed to determine the seroprevalence and congenital infection status of *T. gondii*, rubella, HCV, CMV (IgM and/or IgG), HIV, syphilis, and HBsAg in pregnant women and to inform the development of hospital- and national-level screening algorithms.

## 2. Materials and Methods

### 2.1. Study Design and Data Collection

In this study, test results belonging to a total of 7481 pregnant women aged 15–49 (with the International Classification of Diseases (ICD-10) diagnosis codes Z.33 and Z.34) sent to the Medical Microbiology Laboratory of Sincan Tertiary Hospital between 1 January 2020 and 31 December 2024 were retrospectively examined. The serological results of each pregnant woman at the time of initial application were included in this study. Cases that were only IgM positive, without IgG serological positivity, were not automatically classified as acute infections. They were classified according to the results of a repeat test after 2 weeks. The IgG avidity test was only performed in selected cases, namely, in women with concurrent IgG and IgM positivity for *T. gondii*, CMV or rubella, in order to distinguish recent infections from past infections. Our hospital is located in Ankara, the capital of Turkey, and serves a population of approximately 1 million. It is a 525-bed training and research hospital that treats patients from Sincan Penitentiary, one of Turkey’s largest prisons.

**Figure 1:**
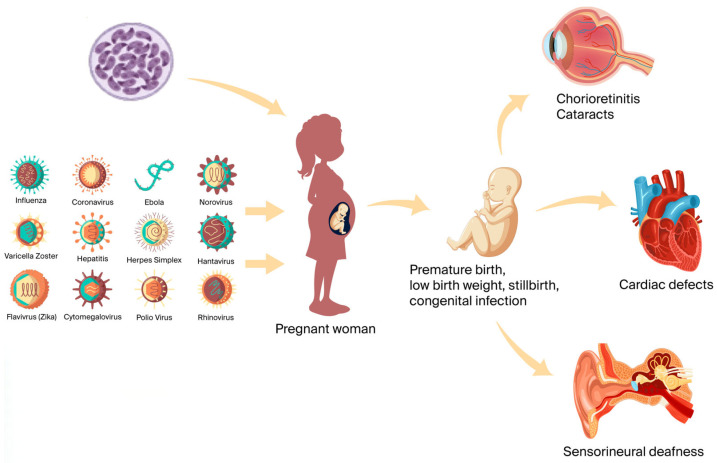
Maternal exposure to TORCH infections during pregnancy and fetal outcomes. Created in BioRender. Akıllı, F. M. (2026) https://BioRender.com/xc0fi0j.

**Figure 2:**
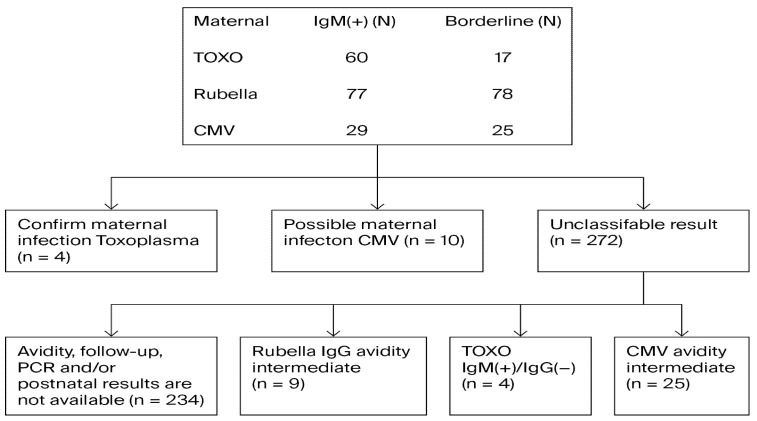
Flow chart of the study for Toxoplasma, rubella, and CMV.

The serological results for syphilis (VDRL, TPHA, and/or FTA-ABS), anti-*T. gondii* IgM and IgG, anti-rubella IgM and IgG, anti-CMV IgM and IgG, HbsAg, and anti-HCV were obtained using the hospital automation system. The study design is represented graphically in Figure 1. Figure 2 shows the workflow diagram for Toxoplasma, rubella and CMV.

This study was approved by the Scientific Research Ethics Committee of Sincan Training and Research Hospital (Decision No: BAEK-2025-91).

### 2.2. Serological Testing of TORCH

Serological tests were performed using a chemiluminescence microparticle immunoassay to detect IgG and IgM antibodies against *T. gondii*, rubella, and CMV.

Blood samples taken during pregnancy were centrifuged and then tested for *T. gondii*-, rubella-, and CMV-specific antibodies using MAGLUMI X3/X6 (Snibe, Shenzhen, China) and Abbott Alinity (Abbott Park, IL, USA) devices in accordance with the manufacturer’s instructions. Nontreponemal tests, including the Rapid Plasma Reagin (RPR) test and the Venereal Disease Research Laboratory (VDRL) test (e.g., Laboquick (Türkiye), One Step (China), Abon (Türkiye), were utilised for the detection of active infection. Samples yielding positive results were subsequently confirmed using treponemal-specific tests, including the *Treponema pallidum* haemagglutination assay (TPHA) or fluorescent treponemal antibody absorption (FTA-ABS). Lipemic and haemolysed sera were excluded from this study, as were repeat samples from the same patient. Cases with missing key serological parameters were excluded from analyses requiring confirmatory testing. *T. gondii* IgM, rubella IgM, and CMV IgM values below 2 AU/mL were considered negative when tested on the MAGLUMI X3/X6 device. Intermediate levels were defined as values of 2–2.6 AU/mL for *T. gondii*, 2–3 AU/mL for rubella, and 2–4.2 AU/mL for CMV.

### 2.3. Statistical Analysis

The pregnant women included in this study were divided into three groups according to their age: 15–24, 25–34, and 35–49. In the statistical analyses, descriptive statistics, percentages, and frequencies were determined for categorical variables. For normal continuous variables, one-way ANOVA was used for age group comparisons. Spearman’s rho and Kendall’s tau-b correlation coefficients were used to analyse age-serological parameter correlation, depending on the data distribution. The chi-squared (χ^2^) test was utilised for the analysis of categorical variables, with post-hoc analyses being conducted for comparisons involving more than two groups. The Bonferroni correction was employed in the post-hoc analysis. In all analyses, the statistical significance level (*p* value) was set at <0.05, and the analyses were performed using the IBM SPSS 26.0 (IBM Corp., Armonk, NY, USA/Canada) software package.

## 3. Results

This study included 7481 pregnant women (mean age: 28.21 ± 5.53 years, range: 15–49 years). Sample sizes varied by test: HBsAg, 7372; anti-HCV, 7165; anti-*T. gondii* IgM, 6135; IgG, 6206; anti-CMV IgM, 6309; anti-CMV IgG, 6300; anti-rubella IgM, 6211; anti-rubella IgG, 6309; IgG, 6081.

Anti-HCV positivity was 0.12% (9/7166), anti-*T. gondii* IgG 16.51% (1027/6207), anti-rubella IgG 95.5% (5809/6081), and anti-CMV IgG 98.63% (6130/6212). Table 1 shows detailed seropositivity percentages of antibodies against the pathogens.

Twenty patients underwent *T. gondii* IgG avidity testing, and four were diagnosed with acute toxoplasmosis. In two pregnant women, IgM was positive while IgG was initially negative; repeat testing at 2-week intervals confirmed acute infection. Two additional pregnant women were positive for both IgM and IgG and were diagnosed with acute infection due to low IgG avidity.

Among 55 women tested for rubella IgG avidity, nine had intermediate values, and 46 had high avidity, with no acute rubella infection detected. The mean age of the IgG-negative group was 28.10 years (95% confidence interval [CI]: 27.95–28.26), and that of the IgG-positive group was 27.46 years (95% CI: 26.12–28.80).

Low CMV IgG avidity was detected in six of 29 women and interpreted as acute CMV infection. Seven women were positive for both CMV IgM and IgG, while 25 had intermediate IgM values. Ten women were diagnosed with acute CMV infection; retrospective records did not indicate whether foeto-maternal complications occurred.

Among women screened for anti-HCV, nine exceeded the cut-off, and three were HCV RNA PCR-positive.

Of 5004 women with VDRL/RPR results, 4991 were negative and 13 (0.2%) were positive. Three of these underwent TPHA testing and were confirmed positive; one experienced a spontaneous miscarriage.

Of 48 HbsAg-positive women, HBV DNA PCR was positive in three with available data. Two were further tested for hepatitis delta virus (HDV), and one was antibody-positive.

The mean age of the HBsAg-negative group was 27.77 years (95% CI: 27.61–27.93) and that of the positive group was 29.70 years (95% CI: 29.28–30.12), with the positive group being older, potentially reaching statistical significance. A significant difference was observed for *T. gondii* IgG between groups (*p* < 0.001). For TOXO IgM, the mean ages were 28.08 years (95% CI: 27.93–28.23) for the negative group, 28.24 years (95% CI: 26.53–29.94) for the positive group, and 27.83 years (95% CI: 23.97–31.70) for the intermediate group, with no significant age differences.

Rubella IgG positivity differed significantly among age groups 15–24, 25–34, and ≥35 years (*p* < 0.001). The mean age of seronegative individuals was 27.58 years (95% CI: 26.64–28.53) and that of seropositive individuals was 28.10 years (95% CI: 27.95–28.25), with no significant age difference.

For CMV IgG, the mean ages were 27.02 years (95% CI: 25.75–28.28) for negatives and 28.09 years (95% CI: 27.94–28.25) for positives. Among individuals with negative, positive, and intermediate results, the mean ages were 28.07 years (95% CI: 27.92–28.23), 27.14 years (95% CI: 24.06–30.22), and 30.39 years (95% CI: 27.72–33.05), respectively. CMV IgG differences were not significant between groups (*p* = 0.054).

The mean age was 28.06 years (95% CI: 27.91–28.22) for HBsAg-negative individuals and 30.86 years (95% CI: 29.16–32.57) for positives, with a significant difference between groups (*p* = 0.009).

For anti-HBs, the mean age was 29.36 years (95% CI: 29.08–29.65) for seronegatives and 27.16 years (95% CI: 27.01–27.31) for positives, with a significant difference between the groups (*p* < 0.001).

For anti-HCV, the mean age was 28.08 years (95% CI: 27.92–28.23) for negatives and 32.20 years (95% CI: 22.61–41.79) for positives, with no significant difference (*p* = 0.247) (Table 2).

For anti-HIV, the mean age of the negative group was 28.08 years (95% CI: 27.93–28.23); insufficient data were available to determine the mean age for the positive group.

## 4. Discussion

This study retrospectively assessed the first-trimester seroprevalence of *T. gondii* IgM/IgG, rubella IgM/IgG, CMV IgM/IgG, syphilis, HIV, and HCV and the frequency of HbsAg in pregnant women in our region to determine the epidemiology of TORCH infections. This study hypothesised that the findings would provide valuable insights into developing national and regional strategies for diagnosing pregnancy-related infections and for monitoring pregnant women in hospital and primary healthcare settings.

In this study, the positivity rates of IgG antibodies were 16.5% (*T. gondii*) (95% CI: 15.6–17.4), 95.5% (rubella) (95% CI: 94.9–96.0), and 98.7% (CMV) (95% CI: 98.3–98.9). The overall seropositivity rates of IgM antibodies were 0.96% (*T. gondii*) (95% CI: 0.73–1.24), 1.2% (rubella) (95% CI: 0.94–1.51), 0.46% (CMV) (95% CI: 0.34–0.73), while HBsAg and anti-HCV positivity were 0.65% (95% CI: 0.52–0.93) and 0.12% (95% CI: 0.06–0.23), respectively. This study included 234 pregnant women whose postnatal foetal outcomes could not be determined through follow-up ultrasonography. A common limitation of all automated systems is the inability to capture pregnancy outcomes through shared medical records.

TORCH infections, especially when contracted during the first trimester of pregnancy, can lead to foetal malformations, miscarriage, or stillbirth through vertical transmission [20,21]. Studies report that the seroprevalence of *T. gondii* antibodies in the normal population ranges from 10% to 90% [20,21,22,23,24,25,26]. Prevalence increases with age and is influenced by multiple factors, including environmental conditions, geographical location, socio-cultural status, climate, transmission routes, community immunity, dietary habits, cat ownership, and health education [13,25,26]. A multicentre study in our country investigated the seroprevalence of *T. gondii*, rubella, and CMV in pregnant women. Kul et al. reported IgG seropositivity of 21% (anti-*T. gondii*), 96.5% (anti-rubella), and 56.2% (anti-CMV) [13]. The findings on *T. gondii* and rubella seropositivity were consistent with those of previous studies conducted in the country. In this study, four of the 20 pregnant women tested for *T. gondii* IgG avidity were diagnosed with acute toxoplasmosis. *T. gondii* IgM was positive in two pregnant women, while IgG was negative. In these patients, acute toxoplasmosis was confirmed based on the detection of *T. gondii* IgM positivity and IgG negativity in serum samples tested at 2-week intervals. *T. gondii* IgM and IgG were positive in two pregnant women, and acute infection was diagnosed based on low *T. gondii* IgG avidity. No association was observed between *T. gondii* infection and pregnancy outcomes such as miscarriage, preterm birth, or stillbirth. This finding is consistent with other studies that also report no association between toxoplasmosis and pregnancy outcomes [11].

The rubella IgG avidity test was performed on 55 patients, revealing high avidity in 46 pregnant women and moderate avidity in nine cases, with no acute rubella infection detected. CMV IgG avidity testing in 29 patients showed low avidity in six cases, confirming acute CMV infection. CMV IgM and IgG positivity was observed in seven pregnant women, while 25 showed moderate CMV IgM levels. During follow-up, 10 pregnant women were diagnosed with acute CMV infection, with no foetal-maternal complications. False positive IgM and or intermediate avidity results may lead to misdiagnosis and unnecessary treatment. In suspicious cases, tests should be repeated at two-week intervals.

Screening for Toxoplasma, rubella, and CMV before or during early pregnancy is crucial for diagnosis and potential intervention. In our country, no standard recommendation or guideline exists regarding preconception or early-pregnancy screening, or which agent to screen for and when, which remains a controversial debate. The prevalence of these infections varies between countries and even regions; therefore, knowing the seroprevalence is essential for developing screening strategies and counseling seronegative women of childbearing age and pregnant women on prevention and vaccination against these infections [27,28,29]. Studies across different regions of Europe report *T. gondii* seropositivity in women of childbearing age ranging from 9.1% to 40.5% [5]. In this study, anti-Toxoplasma IgG, anti-rubella IgG, and CMV seropositivity aligned with these findings; however, limited access to patient records prevented full assessment of foeto-maternal outcomes. However, the inability to access patient records and the inability to adequately determine foeto-maternal outcomes constitute limitations of our study.

Studies conducted in our country report HBsAg seropositivity in pregnant women ranging from 1.2% to 19.2% [16]. Ghazzawi et al. reported positivity of 7.9% (HBsAg), 5.8% (HIV), and 0.3% (HIV/HBV co-infection) among 394 pregnant women in Sierra Leone, with a mean age of 24.4 ± 4.9 years [30]. In our study, HBsAg seropositivity was 0.65% (mean age: 28.21 ± 5.53 years), with positive HBV DNA PCR in 20.8% (10/48). These findings are consistent with previous studies regarding HBsAg seropositivity.

Recently, effective antiretroviral treatment, prevention measures, and awareness programmes have been implemented to reduce the global HIV pandemic. According to 2023 estimates, approximately 40 million individuals are living with HIV, with 1.5 million being children under 15 years [31]. Owing to vertical transmission routes, screening for HIV and HBV remains essential during pregnancy.

In this study, no HIV/HBV co-infection was detected; HIV RNA PCR confirmed negative results in two pregnant women who tested positive for HIV antibodies. Duri et al. investigated HBV and HIV seroprevalence, risk factors, vertical transmission rates, and pregnancy outcomes [32], reporting an overall antenatal HBsAg seropositivity rate of 2.65%, with 1.17% in the HIV-negative group and 4.11% in the HIV-infected group. Despite the gradual increase in HIV prevalence among pregnant women reported in recent studies, continued follow-up and counselling remain essential [32].

National and international guidelines recommend testing HBsAg-positive individuals for HDV [31,33,34,35]. In this study, eight patients underwent HDV reflex testing, with one positive for HDV antibodies, yielding a screening rate of 16.6% (8/48). According to the 2023 update of the Turkish Society of Clinical Microbiology and Infectious Diseases Viral Hepatitis Working Group Consensus Report, HBeAg, anti-HBe, HBV DNA, serum ALT, and other relevant health factors should be assessed in all HBsAg-positive pregnant women [34] Given the shared transmission routes of HBV, HCV, HIV, and HDV, we recommend increased screening for these viruses in pregnant women, in line with the 2030 elimination targets of screening programmes. Despite the recent rise in anti-vaccination rhetoric globally [36,37,38,39], pregnant women should be educated on the importance of vaccination and informed about pathogens without an available vaccine through platforms such as maternity schools and social media. Research shows that vaccinations are crucial. Routine childhood vaccinations have prevented approximately 508 million cases of disease, 32 million hospitalisations, and 1,129,000 mortalities since the 1990s, saving an estimated $540 billion in direct costs and $2.7 trillion in societal costs [38].

Research indicates that HCV affects 0.6–2.4% of pregnancies, with a mother-to-child transmission rate ranging from 8% to 15% [36]. In this study, anti-HCV positivity was detected in 0.1% (9/7166) of cases. Of the six seropositive patients tested, three had HCV RNA levels above the cut-off by PCR. These findings highlight the need to re-evaluate strategies for further HCV RNA testing in anti-HCV-positive patients. We recommend that all individuals with a positive serological test for HCV should undergo reflex testing for HCV RNA.

Syphilis is a sexually transmitted infection caused by *T. pallidum* and can be transmitted from mother to child during pregnancy, leading to adverse outcomes, including low birth weight, premature birth, and stillbirth [18,19,40]. Syphilis and HIV infection have a reciprocal relationship, each facilitating the transmission of the other [40]. Erin et al. report testing 10,449 pregnant women for syphilis using the RPR assay, with a seropositivity rate of 0.19% [35,41]. In this study, VDRL seropositivity was identified in 0.2% (13/5004) of cases; however, no cases of syphilis were confirmed via subsequent TPHA testing. These findings are consistent with those of previous research.

Regrettably, the absence of cost-effectiveness in screening has led to the absence of national guidelines recommending such screenings for pregnant women. This phenomenon is also evident in our own country. However, research has indicated that the risk of TORCH is elevated in regions characterized by limited educational attainment and socioeconomic disadvantage. This finding suggests that the provision of educational resources to women of reproductive age as part of preventive health services may contribute to a reduction in the identified risk [42].

To our knowledge, this study is the first to examine the seropositivity of TORCH infections in pregnant women in our region. The findings highlight the need to strengthen screening and vaccination programmes. The management of TORCH infections in pregnant women should be guided by a consensus among all relevant stakeholders. Interpreting and managing serological results is challenging owing to nonspecific and false-positive IgM results. Furthermore, IgM positivity does not exclude the possibility of reactivation or latent infection. Determining the timing of maternal infection requires a careful clinical history, including consideration of concurrent IgG seroconversion or IgM positivity with low IgG avidity. This assessment should be conducted by an infectious disease or maternal-foetal medicine specialist. Screening for TORCH factors can impose a considerable financial burden on national healthcare systems.

### Limitations

This study has some limitations. First, it was conducted at a single centre. Second, it was retrospective and observational, with incomplete pregnancy data. The study strengths include an adequate sample size. However, the inability to follow up patients with positive TORCH screening results or abnormal ultrasound findings limits the validity of this study, as the outcomes of these cases remain uncertain. This limitation arose largely from the hospital transitioning into a tertiary care facility in 2023, a change that is expected to improve patient follow-up in the future.

## 5. Conclusions

TORCH infections remain a major cause of perinatal morbidity and mortality. The need for routine screening for TORCH agents continues to be widely debated. In this study, high seropositivity rates were observed for CMV and rubella, while the risk of Toxoplasma infection persists. The foeto-maternal complications associated with TORCH infections highlight the need to develop national and regional screening strategies. Promoting vaccination programmes remains critically important. Regarding maternal and foetal health, emphasis should focus on providing pre-pregnancy counselling, enhancing educational resources during pregnancy, and implementing diagnostic procedures to guide treatment decisions in cases of intrauterine infection identified during pregnancy monitoring.

## Figures and Tables

**Table 1:** Seropositivity rates of *T. gondii*, rubella, CMV, HBsAg, and HCV in pregnant women.

Test	Positive N (%)	95% CI	Borderline N (%)	Negative N (%)	Total N
Anti CMV IgG	6130 (98.69)	98.3–98.9	-	81 (1.3)	6211
Anti CMV IgM	29 (0.46)	0.34–0.73	25 (0.4)	6246 (99.1)	6300
Anti HCV	9 (0.12)	0.06–0.23	-	7157 (99.87)	7166
HBsAg	48 (0.65)	0.52–0.93	-	7324 (99.3)	7372
HIV	2 (0.02)	0.00–0.07	-	7144 (99.9)	7146
rubella IgG	5809 (95.5)	94.9–96.0	-	271 (4.5)	6080
rubella IgM	77 (1.2)	0.94–1.51	78 (1.2)	6160 (97.5)	6315
TOXO IgG	1027 (16.5)	15.6–17.4	-	5177 (83.4)	6204
TOXO IgM	60 (0.96)	0.73–1.24	17 (0.27)	6153 (98.7)	6230

CMV, cytomegalovirus; HCV, hepatitis C virus; HBsAg, hepatitis B surface antigen; HIV, human immunodeficiency virus; TOXO, Toxoplasmosis; IgG, immunoglobulin G; IgM, immunoglobulin M.

**Table 2:** Distribution of serological test results by age group and test type.

Age Group	Test	Positive n (%)	Borderline n (%)	Negative n (%)	Total n (%)	*p*
15–24 years	rubella IgM	25 (32.5)	28 (35.9)	1727 (28)	1780 (28.2)	
	rubella IgG *	1610 (27.7)	-	104 (38.4)	1714 (28.2)	
	CMV IgM	8 (27.6)	8 (32)	1759 (28.2)	1775 (28.2)	
	CMV IgG	1716 (28.0)	-	32 (39.5)	1748 (28.1)	
	TOXO IgM	19 (31.7)	5 (29.4)	1733 (28.2)	1757 (28.2)	
	TOXO IgG ^+^	223 (21.7)	1 (50)	1519 (29.3)	1743(28.1)	
	Anti-HCV	2 (22.2)	-	1994 (27.9)	1996 (27.9)	
	Anti-Hbs	788 (24.2)	-	790 (33.4)	1578 (28.1)	
	HbsAg	4 (8.3)	-	2047 (27.9)	2051 (27.8)	
25–34 years	rubella IgM	42 (54.5)	43 (55.1)	3599 (58.4)	3684 (58.3)	
	rubella IgG *	3422 (58.9)	-	118 (43.5)	3540 (58.2)	0.001
	CMV IgM	18 (62.1)	13 (52)	3642 (58.3)	3673 (58.3)	
	CMV IgG	3576 (100.0)	-	42 (51.9)	3618 (58.3)	
	TOXO IgM	34 (56.7)	9 (52.9)	3586 (58.3)	3629 (58.3)	
	TOXO IgG ^+^	573 (55.8)	-	3045 (58.8)	3618 (58.3)	<0.001
	Anti-HCV	4 (44.4)	-	4161 (58.1)	4165 (58.1)	
	Anti-Hbs ^#^	2325 (71.4)	-	961 (40.6)	3286 (58.4)	0.001
	HbsAg ^&^	34 (70.8)	-	4251 (58)	4285 (58.1)	0.009
≥35 years	rubella IgM	10 (13)	7 (9)	834 (13.5)	851 (13.5)	
	rubella IgG *	777 (13.4)	-	49 (18.1)	826 (13.6)	
	CMV IgM	3 (10.3)	4 (16)	845 (13.5)	852 (13.5)	
	CMV IgG	838 (13.7)	-	7 (8.6)	845 (13.6)	
	TOXO IgM	7 (11.7)	3 (17.6)	834 (13.6)	844 (13.5)	
	TOXO IgG ^+^	231 (22.5)	1 (50)	613 (11.8)	845 (13.6)	
	Anti-HCV	3 (33.3)	-	1001 (14)	1004 (14)	
	Anti-Hbs	145 (4.5)	-	616 (26)	761 (13.5)	
	HbsAg	10 (20.8)	-	1026 (14)	1036 (14.1)	

^&^, *: A difference arose between group 1 and group 2. ^#^, ^+^: A difference arose between group 2 and group 3. IgM, immunoglobulin M; IgG, immunoglobulin G; CMV, cytomegalovirus; TOXO, toxoplasmosis; HCV, hepatitis C virus; HBsAg, hepatitis B surface antigen.

## Data Availability

The raw data supporting the conclusions of this article will be made available by the corresponding author upon request.

## Abbreviations

The following abbreviations are used in this manuscript:

HBV	Hepatitis B virus
HIV	Human immunodeficiency virus
HCV	Hepatitis C virus
CMV	Cytomegalovirus
WHO	World Health Organization
ICD-10	International Classification of Diseases, 10th Revision
VDRL	Venereal Disease Research Laboratory test
TPHA	Treponema pallidum hemagglutination assay
FTA-ABS	Fluorescent Treponemal Antibody Absorption test
TORCH	*Toxoplasma gondii*, Other infections, rubella, Cytomegalovirus, Herpes Simplex Virus
